# Spectroscopic photoacoustic/ultrasound/optical-microscopic multimodal intrarectal endoscopy for detection of centimeter-scale deep lesions

**DOI:** 10.3389/fbioe.2023.1136005

**Published:** 2023-01-26

**Authors:** Jinsheng Jiang, Chuqi Yuan, Jiaxi Zhang, Zhuojun Xie, Jiaying Xiao

**Affiliations:** Department of Biomedical Engineering, School of Basic Medical Science, Central South University, Changsha, China

**Keywords:** spectroscopic imaging, photoacoustic endoscopy, deep lesion, indocyanine green, multimodal

## Abstract

The inadequacy of existing colorectal imaging tools has significantly obstructed the efficient detection of colorectal cancer. To address this issue, this work presents the cross-scale endoscopic imaging of rectal tumors with a combined photoacoustic/ultrasound tomography system and wide-field optical microscopy. This multimodal system combines the merits of centimeter-scale deep penetration, multi-spectral imaging, cross-scale imaging ability, low system cost, and 360° view in a single modality. Results indicated that the proposed system could reliably depict the location of the cancer invasion depth spectroscopically with indocyanine green The tumor angiogenesis can be well identified in the wide-field optical imaging mode, which helps to localize the tumors and guide the following photoacoustic/ultrasound scan. This work may facilitate the accurate characterization of colorectal cancer and promote the clinical translation of photoacoustic-based colorectal endoscopy.

## 1 Introduction

Colorectal cancer is the third most common malignant tumor after lung cancer and breast cancer. It has an annual global incident rate of about 1.4 million, and its incidence and mortality rates have increased globally over the past 10 years (Mármol et al., 2017; Dekker et al., 2019).

In current practice, colonoscopy is the primary basis for the diagnosis of colorectal cancer (Ho et al., 2018), which provides the position and high-definition morphology of the lump on the colon’s inner surface. Due to the lack of depth-resolved information, following colon biopsy is often required for determinative diagnosis, which is time-consuming and low efficiency. Routing radiologic colorectal imaging modalities (Akasu et al., 2009; Ho et al., 2018; Dekker et al., 2019), including endoscopic ultrasound (EUS) (Garcia-Garcia et al., 2010; Vikhareva Osser and Valentin, 2011), barium enema X-ray, CT colonography (CTC), magnetic resonance (MR) (Akasu et al., 2009), and positron emission tomography (PET), have high penetrations. Still, they are generally limited in functional imaging ability, making them insensitive to subtle cancer-related abnormalities in the colon wall (Morris and Perkins, 2012; Ho et al., 2018). Therefore, there’s still solid demand for exploring new high-penetration colorectal imaging technologies, which can act as a more effective complementary technique to colonoscopy for enhanced diagnostic and therapeutic procedures for colorectal cancer.

Photoacoustic imaging (PAI) is a promising tool for the non-invasive detection of cancer (Wang and Hu, 2012; Attia et al., 2019). As a hybrid imaging method of optical excitation and ultrasound detection, PAI combines the rich contrast of optical imaging with the high resolution and deep penetration of US imaging. It ultrasonically breaks the optical diffusion limit, enabling focused imaging of deep tissues with spatial resolutions much higher than existing pure optical imaging technologies. As the near-infrared laser commonly employed in PAI is mainly absorbed by the hemoglobin in the tissue, PAI is highly sensitive to abnormal angiogenesis, which is a prominent sign of cancer (Huang et al., 2020). Besides, because PAI contrast relies on optical absorption, a wide variety of bio-compatible exogenous contrast agents are available for PAI (Ntziachristos, 2010; Yu et al., 2018; Zeng et al., 2022). By passive, active, or triggered targeting strategies, PAI can take full advantage of the optical absorption characteristics of these contrast agents and exhibit many merits in various tumor detection (Zhou et al., 2019), including rich contrast and function, improved sensitivity and specificity, and extended imaging depth. Therefore, PAI may become a powerful tool for colorectal cancer diagnosis in the embodiment of endoscopy (Zhao et al., 2019; Guo et al., 2020), which promises extraordinary flexibility and high contrast than conventional radiologic modalities.

Photoacoustic endoscopy (PAE) can be versatile in system design. Since the invention of the prototype PAE probe with integrated optical illumination units, PAE has made substantial progress, notably in aspects of probe diameter, scanning speed, and spatial resolution. However, these achievements, to some extent, come at the expense of image depth and multi-spectral imaging ability. Most current single-element-based small PAE probes use a 532 nm high-repetition laser with pulse energy lower than 0.3 mJ (Yang et al., 2009; Yang et al., 2012a; Yang et al., 2012b; Li et al., 2014; Yang et al., 2015), which offers high-quality rendering of the blood vessel networks in the superficial tissue layers but within a penetration depth of only approximately 1 mm. In contrast, although array-based PAE probes have high-penetration and spectroscopic imaging ability with high-pulse-energy tunable optical parametric oscillator (OPO) lasers, they are costly expensive, generally low in central-frequency and lateral resolution, and big in size (2.5 cm on average) which is due to the complicated structural constraints of optical illumination and ultrasound detection (Yuan et al., 2010; Wang et al., 2021b). Hence, their application is limited in general biomedical studies.

In this study, we constructed a novel acoustic-resolution-based photoacoustic/ultrasound endoscopic (AR-PA/USE) system with a centimeter-scale imaging depth and integrated it with wide-field optical endoscopy. We preliminarily explored its potential in colorectal cancer detection. We tested the system with field-test experiments, simulations, phantom experiments, and *in vivo* experiments. Results indicate that the proposed multi-functional endoscopic system can provide comprehensive information and potentially contribute to diagnosing lesions in the rectum and surrounding organs.

## 2 Materials and methods

### 2.1 Imaging system setup

**FIGURE 1:**
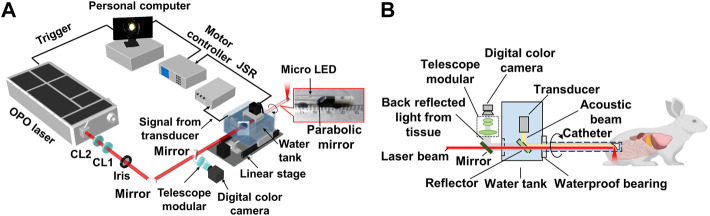
System setup of the multimodal AR-PA/USE and wide-field optical imaging system. **(A)** Schematic illustration of the system; **(B)** Illustration of the system’s laser incidence, ultrasound detection, wide-field optical endoscopic imaging schemes, and a photograph of the probe’s distal end. CL1, positive cylindrical lens; CL2, negative cylindrical lens.

Figure 1A illustrates the schematic of the multimodal imaging system. Pulsed excitation light from a 532 nm pumped optical parametric oscillator (OPO) laser (SpitLight OPO 600 mid-band, Innolas, Krailling, Germany) passed through a set of cylindrical focus lenses and iris for spatial filtering and shaping, then was delivered through an optical window of the water tank and entered the probe for the spiral endoscopic scan. The repetition rate of the OPO laser was 20 Hz, and its pulse energy was basically controlled at 8 mJ/cm^2^ on the sample. The probe housing was transparent polyethylene, about 15 cm in length, with an outer diameter of 8 mm and a wall thickness of 0.5 mm. A protected aluminum-coated parabolic mirror (#37–282, Edmund Optics, NJ, 12.5 mm focal length) was mounted at the distal end of the probe for reflection of the excitation laser and collection of the ultrasound. The collected ultrasound by the parabolic mirror was reflected by a 45º CaF2 reflector to a 10 MHz planar ultrasonic transducer, as shown in Figure 1B. For wide-field optical endoscopic imaging, a white light micro-LED of about 1 mm in size was placed near the parabolic mirror for illumination, and a collimated digital color camera with a telescope modular was employed for full-field optical image collection. The probe was driven by a stepping motor with a belt-gear set for the sectoral endoscopic scan. A pulser/receiver (DPR500, Imaginant, NY) was employed to generate pulsed ultrasound in ultrasound imaging and amplify the collected photoacoustic/ultrasound signals. The final was digitalized with a data acquisition card (NI PCI-5124, 100 Ms/S, 12-Bit, National Instruments Corporation, TX) and stored in the computer for future processing. The whole system was synchronized by the laser and controlled by a LabView program.

### 2.2 Image reconstruction method

Conventional PAE assumes that the acoustic wave travels in a thin straight line during the spiral scanning, thus in the image reconstruction, the pixel value for an arbitrary pixel at (*r,θ*) in polar coordinates is given by the direct back aligning of the collected photoacoustic signals, which is the conventional B-mode method:

$$I\left(r,\theta \right)=S\left(\theta ,\left|r+L\right|/v\right)$$ (1)

Here *S(θ,t)* is the PA signal received when the transducer position is at angle θ and time t, v is the acoustic velocity in the media, and L is the distance from the rotation center to the transducer detection surface. However, this reconstruction method has decreased lateral resolution in the off-focal regions. This work employs an improved back-projection (IBP) method to overcome this issue (Wang et al., 2018). It assumes that performing the spiral scan with the parabolic reflection mirror is equivalent to scanning a virtual point-focused transducer of the same numerical aperture (NA) around the probe axis. This method divides the surface of the virtual point-focused transducer into Np point detectors and gives the reconstructed pixel value with the following equation:

$$I\left(r\right)=\overset{Nd}{\underset{i=1}{\sum }}\overset{Np}{\underset{k=1}{\sum }}A\times S\left(\theta_{i},\left|R_{i,k}-r\right|/v\right)$$ (2)

Here, *θ*_*1*_, *θ*_*2*_, … , and *θ*_*Nd*_ are the angular scan positions, and *R*_*i,k*_ donates the *k*th point detector when the transducer is at angular position *θ*_*i*_. The coefficient *A* weighs the contributions of photoacoustic signals from different transducer positions to the pixel. This algorithm has been probed to improve the lateral resolution and signal-to-noise ratio (SNR) in the off-focal regions. For ultrasound image reconstruction, the acoustic velocity is set to be *v*/2 in Eqn. 1, Eqn. 2 compensating for the two-way acoustic round trip.

### 2.3 Fields test of the imaging system

Conventional PAE assumes that the acoustic wave travels in a thin straight line during the spiral scannin The field characteristics of the proposed system were tested with a 50-μm-thick tungsten wire for both photoacoustic and ultrasound imaging. The tungsten wire was scanned over ten different imaging depths, acting as a point target. During the photoacoustic test, the tungsten wire was illuminated by a multimode fiber with a 3 mm core size, which was scanned along with the tungsten wire to keep the same photoacoustic intensity. The acquired photoacoustic/ultrasound images at different depths were overlaid to produce one final image, and the full-width at half maximums (FWHMs) were acquired as lateral resolutions. Furthermore, the experimental results were compared with the simulation results, where the forward data were generated with a Rayleigh integral method described elsewhere (Luo et al., 2021).

### 2.4 *In vivo* and *ex-vivo* experiments

In the *in-vivo* experiments, four male white New Zealand rabbits (approximately 2 kg) were imaged. The rabbits were kept in the lab for 48 h for acclimatization and fasted for 24 h before the experiment. Each rabbit was first administered a rectal enema with normal saline, then fastened on a steel shelf, after which the probe was inserted into the rabbit’s rectum. The rabbits were maintained under gas anesthesia, with a dose of 2% isoflurane and a flow rate of about 1 L/min. A dose of 0.5 ml indocyanine green (ICG, 2.5 mg/ml) was injected into the left hip of a rabbit, and simultaneous 2D photoacoustic and ultrasound images were acquired at different wavelengths. The wide-field optical endoscopy can monitor the rectum’s inner surface and ensure the acoustic contact of the probe. All the rabbits survived after the experiments.

In the *ex-vivo* experiment, we injected the VX2 tumor cell line (with a concentration of 10^7/ml, in 1.5 ml PBS solution) deep in between the hind leg muscles of the rabbit to generate cancer. The tumor growth was monitored for 14 days until it grew to about 2 cm. Then the rabbit was sacrificed to get the tumor tissue. The rabbit VX2 tumor model has been used widely to study many human cancers. It is a leporine anaplastic squamous cell carcinoma characterized by rapid growth, hypervascularity, and facile propagation in the skeletal muscle (Parvinian et al., 2014). The tumor tissue was finally stained with ICG and buried in a phantom for endoscopic imaging, which had a scattering coefficient of 1/mm and an absorption coefficient of 0.007/mm. We employed 10 to 12 rabbits during this study, and all the rabbits survived after the experiments. All animal experiment procedures followed the instructions of the Department of Laboratory Animals of Central South University (No. 2020KT-39).

## 3 Results

### 3.1 Field tests

**FIGURE 2:**
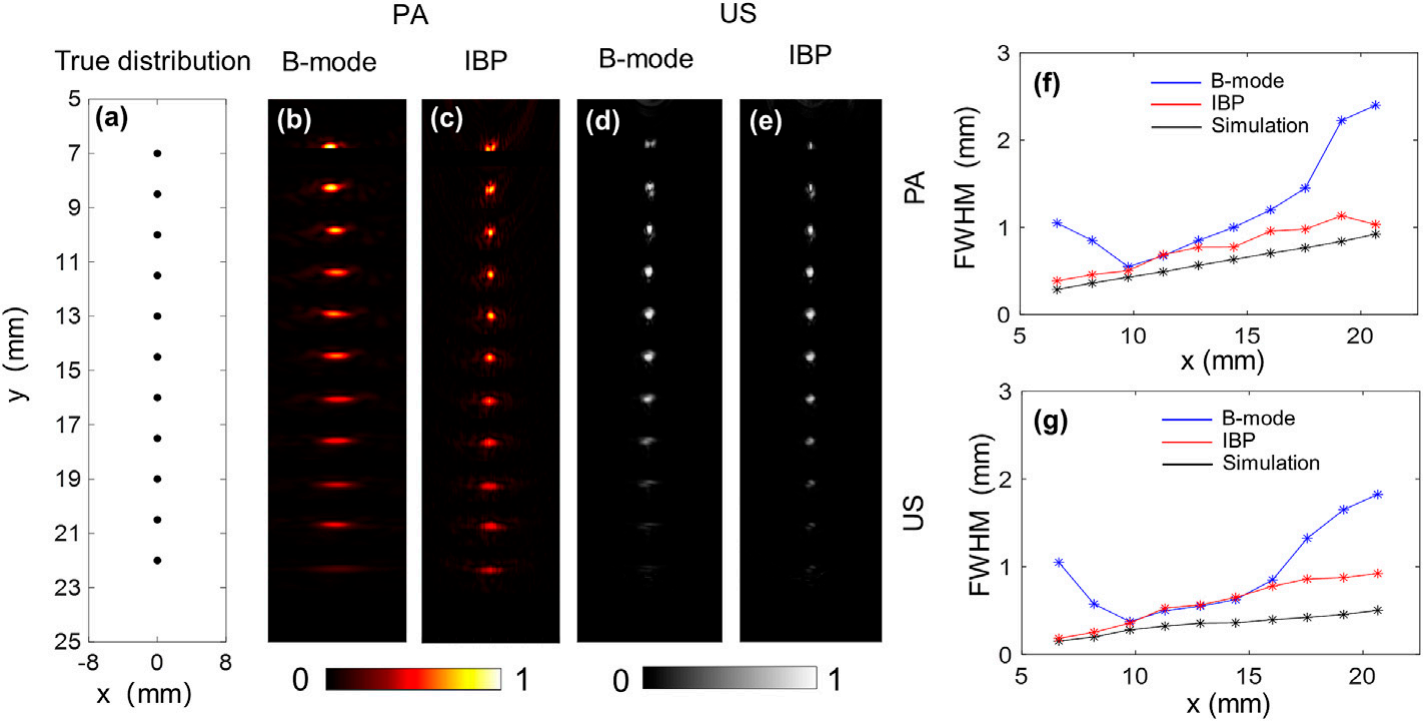
Field test results show lateral resolution changes at different depths of the proposed system, acquired with a point target. **(A)** The true distribution of the point targets; **(B)** and **(C)** are the photoacoustic images of the point target by the B-mode and IBP methods, respectively; **(F)** Obtained FWHMs at different depths from **(B)** and **(C)**. The blue and red lines were calculated from **(B)** and **(C)**, and the black line is the simulation results; **(D)**, **(E)**, and **(G)** are the corresponding results for ultrasound imaging. The maximum amplitudes of the PA and US images are normalized for facilitated comparison.

Figure 2A shows the true distribution of the targets and the coordinates. Figures 2B, C show the acquired photoacoustic images of the metal wire at different depths, and Figure 2F shows the change in the lateral resolution with the image depth. Figure 2D, E, G, depict the corresponding ultrasound imaging results. As expected, the IBP method yields superior lateral resolutions to the conventional method. Results show an increasing trend in the lateral resolution for both photoacoustic and ultrasound imaging. This is because the relative NA of the probe decreases as the target goes further in the axial direction. The photoacoustic lateral resolution was 0.39 mm at the 6.6 mm depth, which grows to 1.03 mm at 20.6 mm. For ultrasound imaging, it was 0.185 mm at 6.6 mm, and 0.96 mm at 20.6 mm. The black lines in Figures 2F, G represent simulated results when the virtual detection surface has similar acoustic parameters to the parabolic mirror. Here, the central frequency was set as 9 MHz, as measured from the experimental data; the effective diameter of the mirror was set to 5.5 mm, and the focal length f was 12.7 mm. Results show that the measured lateral resolutions are close to the theoretical results for photoacoustic imaging. For ultrasound imaging, the measured values were one time larger than the simulated values, possibly because the PE tube decreased the equivalent size of the parabolic mirror. Overall, these results indicate the high spatial resolution of the proposed system over the centimeter-level detection depth.

### 3.2 *In vivo* tracking of the ICG indicator

**FIGURE 3:**
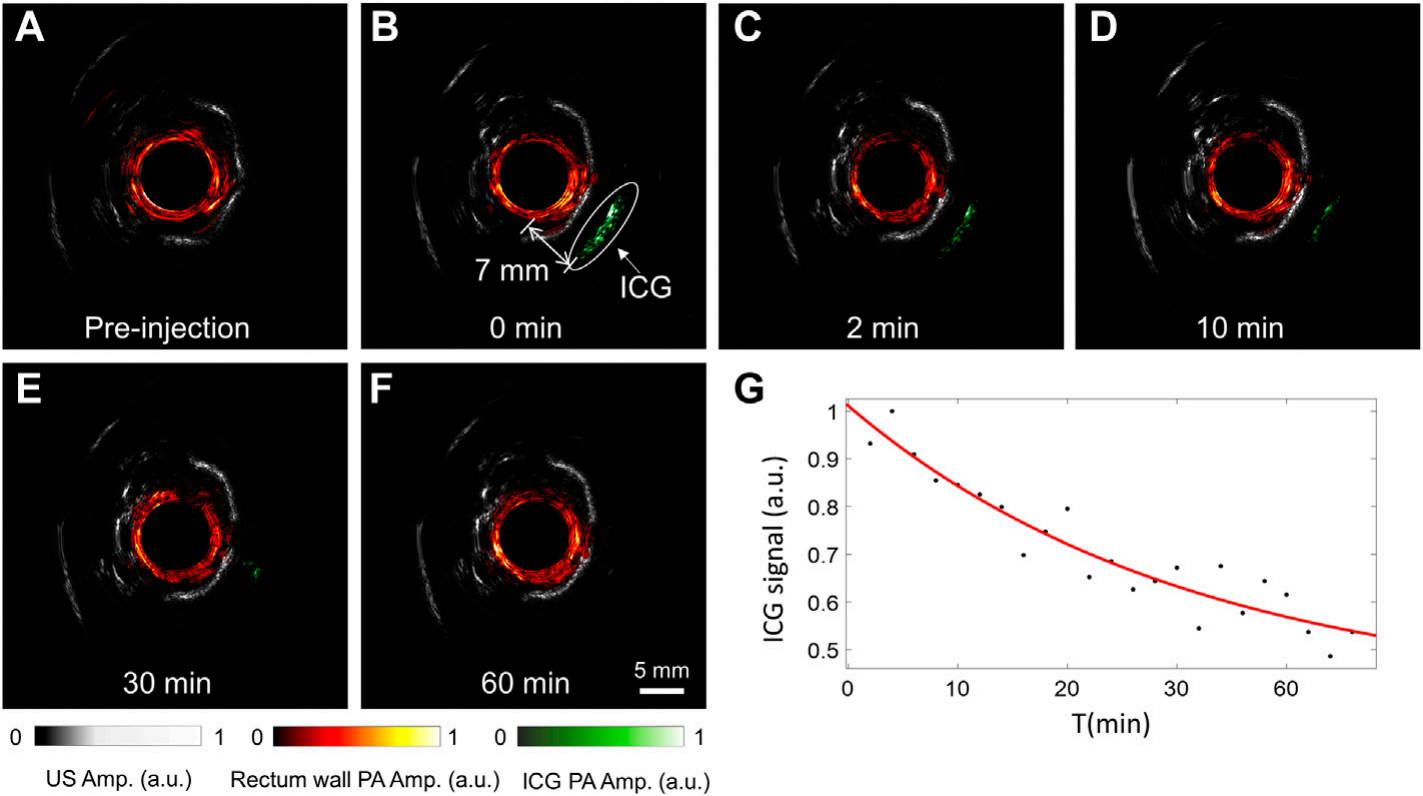
Experimental results for tracking the injected ICG near the rabbit rectum. **(A**–**F)** Comparison of photoacoustic and ultrasound images of the rabbit rectum before **(A)** and after **(B–F)** ICG injection, with the image acquisition time indicated. **(G)** The change of the peak ICG signal with time. The drug injection time was set to 0.

As an FDA-approved dye, ICG is widely used as a photoacoustic contrast agent in basic biomedical and pre-clinical studies for improved sensitivity in cancer detection. Figure 3 shows a group of representative results of photoacoustic images of the rabbit rectum after the ICG injection, which are overlaid onto the ultrasound images. The photoacoustic excitation wavelength was selected to be 800 nm, near the absorption peak of ICG in the blood. Because the outer diameter of the probe is 8 mm, image reconstruction is not performed within this central region. The ultrasound images clearly show the pelvic bones and tendons, which helps the understanding of the anatomic and the acquired photoacoustic images. It is seen that the injected ICG can be well separated from the rectum wall in the photoacoustic images, which was about 7 mm from the rectum wall. While the contrast of ICG is high in the picture, we see no other structures beyond the rectum wall, so the ICG’s signal was easily picked and rendered in green. As the ICG diffused in the tissue with time, the ICG’s signal peak decreased, as seen in Figure 3G. These results demonstrate the superior photoacoustic imaging depth and indicate that the proposed multimodal endoscopic system can reliably map the ICG distribution for cancer detection.

### 3.3 Multi-spectral photoacoustic/ultrasound imaging

**FIGURE 4:**
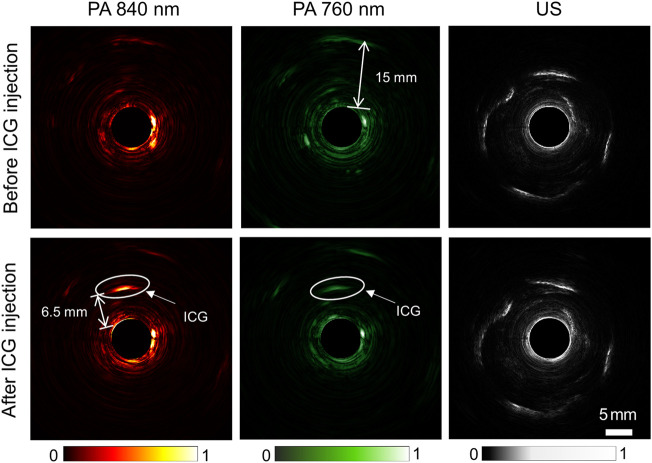
Comparison of photoacoustic and ultrasound images of the rabbit rectum before (top row) and after (bottom row) ICG injection. From left to right, the three columns depict photoacoustic imaging results with 840 nm and 760 nm wavelengths and ultrasound imaging results, respectively.

Existing PA/USE systems generally use a single wavelength of 532 nm, and the imaging depths are generally low, which is not optimal for functional imaging and cancer detection in deep tissues. To explore the potential of our built large-depth multi-wavelength AR-PA/USE system, we compared the imaging results of the same cross-section before and after the ICG injection, as seen in Figure 4. Here, the top row depicts the results before ICG injection and the bottom row shows the results after the ICG injection. From left to right, the three columns display the photoacoustic images for 840 and 760 nm wavelengths and the ultrasound imaging results, respectively. Herein, the 760 nm wavelength served to visualize the concentration of deoxyhemoglobin (Hb), which dominates the optical absorption at this wavelength in biological tissue. The 840 nm wavelength was used to image the concentration of oxygenated hemoglobin (HbO_2_). The photoacoustic signal of ICG approximately 6.5 mm from the probe surface is distinguishable, which shows high absorption in photoacoustic images of both wavelengths, as indicated with white circles. Besides the ICG photoacoustic signal, other photoacoustic and ultrasound signals remain almost unchanged after the ICG injection. This implies our system’s high stability and good capability for deep tumor imaging with the ICG as a contrast agent.

### 3.4 Functional and multimode imaging results

**FIGURE 5:**
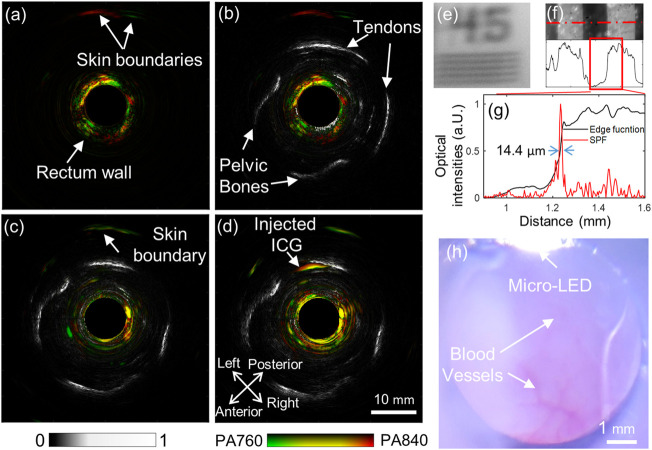
Multi-functional endoscopic imaging results of the rabbit rectum. **(A)** Fused photoacoustic image of 760 and 840 nm; **(B)** Fused dual-wavelength photoacoustic and ultrasound image; **(C)** and **(D)**, fused dual-wavelength photoacoustic and ultrasound images before and after a dose injection of ICG, respectively; **(E)** and **(F)** are the acquired wide-field optical endoscopic image of a positive resolution test target plate, with patterns of 45 circle/mm and 1.0 circle/mm, respectively; **(G)** Edge function (black) and derived PSF (red) of the wide-field optical endoscopy, obtained from the data along the dotted red line in **(F)**; **(H)** The wide-field optical image of the rectum inner surface.

The photoacoustic images acquired under the two different wavelengths can be merged in HSV mode to produce a functional image representing the total hemoglobin concentration and blood oxygen saturation, as described in (Wang et al., 2021a). Figure 5A depicts the merged image with the two single-wavelength photoacoustic images for one rabbit. Herein, the green color suggests a significantly higher photoacoustic intensity at 760 nm than 840 nm, indicating low oxygen saturation. Similarly, red marks a high oxygen saturation. Figure 5B is the overlaid image of the dual-wavelength photoacoustic image (the red-green colormap) with the background of the ultrasound image (the gray colormap). The ultrasound image can visualize continuous boundaries of some pelvic bones and tendons. The photoacoustic image mainly shows the rectal wall (about 2–3 mm thick), which may be due to the existence of dense blood vessels. Figures 5C, D are the merged functional photoacoustic/ultrasound images before and after the ICG injection, respectively, produced from images in Figure 4. With the guidance of the ultrasound image, the ICG injection position revealed by the photoacoustic signals can be better localized; Figure 5E shows one representative image from wide-field optical microscopy. This image clearly shows the inner surface of the rabbit rectum, where some small blood vessels can be well distinguished, as indicated by white arrows. The micro-LED in the probe housing wall can also be seen. This imaging mode acts as a conventional clinical endoscopic diagnostic method. It can guide the AR-PA/USE scan, as the scanning speed of AR-PAE is considerably limited by the low repetition rate of the laser; Figure 5F shows that the system can distinguish a 45 circles/mm pattern from a resolution test target plate (R2L2S1P1, Thorlabs Inc.). The 1.0 circle/mm pattern was images to test the spatial resolution, and its corresponding intensity profile was extracted as the edge function. After differentiating this curve, the system’s point spread function (PSF) is obtained. The spatial resolution of the system was adopted as the FWHM of the PSF and measured at about 14.4 μm. These results indicate the system’s ability for photoacoustic functional and cross-scale imaging, which offers improved diagnostics with comprehensive information.

### 3.5 *Ex-vivo* tumor imaging results

**FIGURE 6:**
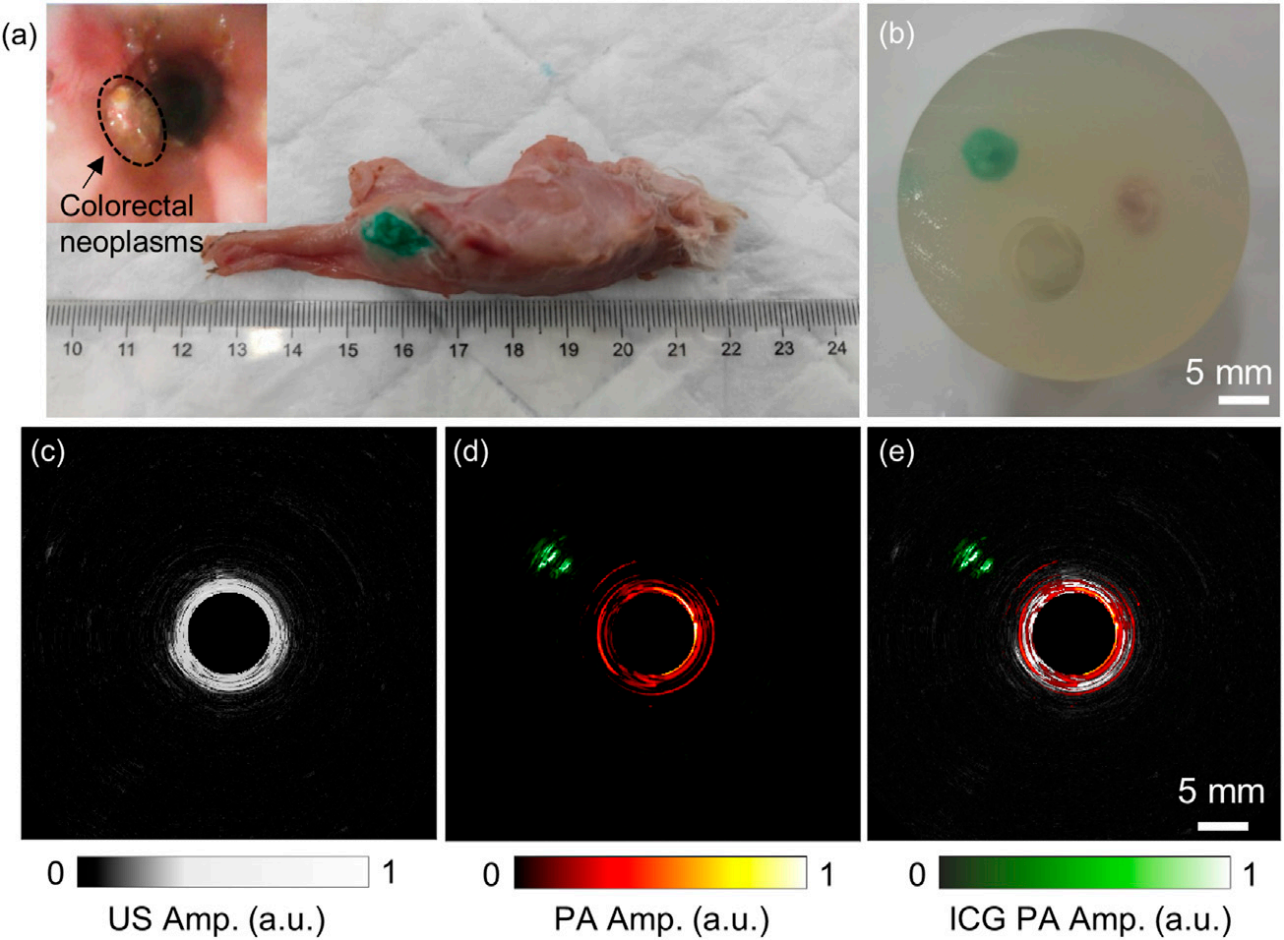
*Ex-vivo* endoscopic imaging results of the ICG-labeled rabbit VX2 tumor. **(A)** The photo image of the excised rectum and the tumor injected with ICG. The small picture in the top left corner shows the endoscopic image of the tumor *in situ*. **(B)** The phantom containing both the tumor and a piece of normal tissue; **(C)** and **(D)** show the obtained ultrasound and photoacoustic images of the phantom. **(E)** The fused photoacoustic and ultrasound image of the phantom. The laser wavelength of the photoacoustic image was 800 nm.

This *ex-vivo* endoscopic imaging experiment preliminarily demonstrated the AR-PA/USE system’s ability to detect colorectal cancer if a deep-seated tumor was targeted with a high-specific contrast agent. Figure 6A shows the harvested rectum from the rabbit. The tumor was identified and stained by local injection of ICG. During the 2 weeks, we tracked and observed the tumor growth with a forward optical endoscopic probe, as seen in the small inset in the top left corner of Figure 6A. Because it is difficult to scan this *ex-vivo* tissue with our system without motion artifacts, we harvested both the ICG-labeled tumor and a piece of normal tissue and buried them about 15 mm deep to demonstrate the high-penetration and high-specific imaging with our built system, as seen in Figure 6B. The acquired ultrasound image is shown in Figure 6C, where the reflection from the phantom inner wall can be seen. However, the reflection from either the tumor or the normal tissue could not be distinguished, which may be because their acoustic impendence is close to the background. Figure 6D is the photoacoustic image at 800 nm, where the ICG-labeled tumor (rendered in green) and the phantom inner wall (red) were visualized, but the normal tissue was not seen. Figure 6E shows the overlaid photoacoustic image on the ultrasound image. These results indicate that while endoscopic ultrasound may not reliably detect some lesions due to its low acoustic contrast of the soft tissues, AR-PAE may serve as a promising tool for high-penetration tumor detection.

## 4 Discussion

Colorectal endoscopic imaging is critical for the clinical management of colorectal cancer. However, current routing colorectal imaging probes such as colonoscopy and EUS either have poor penetration depth or limited molecular/functional imaging abilities. The development of novel colorectal endoscopic imaging modalities may help the accurate characterization of colorectal structures and the detection of dysfunctions, enabling enhanced diagnostic and therapeutic procedures for colorectal diseases. PAE combines the merits of rich optical molecular contrast and high ultrasound penetration, which is highly potential for colorectal cancer diagnosis. In this work, we have demonstrated that the proposed AR-PAE system has high sub-millimeter spatial resolutions and centimeter-scale penetration depth; it can monitor the diffusion of the injected ICG near the rabbit rectum and distinguish the rabbit rectum wall, the surrounding tissues, and the ICG spectroscopically; this system can also precisely locate the ICG-labeled tumor at about a centimeter depth. All these results indicate that the high-penetration spectroscopic AR-PAE may become a reliable tool for colorectal cancer diagnosis.

From the perspective of image information complementarity, PAE is easily combinable with various other endoscopic imaging modalities to provide high-resolution anatomical and rich functional information on biological tissues. Combined PAE and ultrasound endoscopy are conveniently realized by employing the same transducer for ultrasound pulse excitation and detection, which provides both volumetric functional and anatomical information. In addition to ultrasound endoscopy (USE), numerous pure optical imaging modalities such as optical coherence tomography (OCT) (Yang et al., 2011; Dai et al., 2015; Mavadia-Shukla et al., 2018), hyper-spectral imaging (HSI) (Liu et al., 2018), fluorescence imaging (Shao et al., 2012), and wide-field optical imaging (Li et al., 2020) have also been combined with PAE to provide additional optical contrast to the inner surface of the organs. This work integrated the AR-PAE system with EUS and wide-field microscopy, the most used tools for colorectal disease diagnosis. We showed that the EUS could depict the pelvic bones and tendons, helping the understanding of the acquired photoacoustic images. As the scanning of EUS is much faster than AR-PAE, EUS can also help to find the regions of interest to guide the photoacoustic scan. The wide-field optical microscopy can observe subtle physiological changes in the shallow tissue layers, such as the morphology and density of the capillary bed and small blood vessels, as well as the oxygen saturation. In this manner, the observation of the tissue surface and the approximate location and outline description of the deep lesions can be simultaneously realized for in-depth diagnosis.

Although we have preliminarily demonstrated the potential of the proposed multimodal PA/US/optical endoscopic probe for colorectal cancer detection with appropriate contrast agents such as ICG, there are still many technical issues to be addressed to realize the benefits of AR-PAE in clinical settings. First, the proposed system is in rigid form and based on free-space laser delivery, which hinders numerous clinical needs. Therefore, we aim to build a flexible AR-PA/USE catheter that integrates a small step rotator for scanning in the distal end and an imaging fiber bundle for both excitation light delivery and wide-field optical imaging. Second, one major limitation of our proposed method was the long scanning time, mainly due to the low laser repetition rate. In the following studies, we will only perform the photoacoustic scan in selected regions of interest under the guidance of EUS and wide-field optical endoscopy to reduce the scanning time to allow the practical 3D scan. Third, we will try to reveal more deep organs and structures with the help of appropriate contrast agents for photoacoustic imaging. In addition, we used a 10 MHz ultrasound transducer to balance the system’s spatial resolution and penetration depth, but the central frequency was slightly low. In future studies, we will consider increasing the transducer’s center frequency to reconstruct layer structures better (He et al., 2018). Finally, the improved back-projection algorithm used in this study was only an approximate algorithm and cannot guarantee the best dynamic focusing effect. We will develop advanced model-based optimization algorithms to improve further the image quality, which can also help reduce the number of transducer angular positions in the scanning to reduce the data acquisition time.

## 5 Conclusion

We have designed and built an AR-PA/USE system with centimeter-level imaging depth. Using a transducer with a central frequency of 10 MHz, the lateral resolutions of the system at 19 mm depth reached approximately 1 mm for both photoacoustic and ultrasound endoscopic imaging modes. *In vivo* imaging results of the rabbit’s rectum yielded the structure of the rectum and surrounding tissues by ultrasound imaging and provided physiological and functional information by multi-wavelength photoacoustic imaging, such as specific contrast agent distribution and blood oxygen saturation. The integration with wide-field optical endoscopy also enables the system to monitor the rectum surface, provide valuable complemental diagnostic information, and guide the deep-penetration PAE/USE scan. All these results indicate the potential of this high-penetration multi-functional AR-PA/USE system in contributing to the clinical diagnosis of colorectal cancer and rectum-related diseases.

## Data Availability

The raw data supporting the conclusions of this article will be made available by the authors, without undue reservation.
